# Nitrogen Addition Regulates Soil Nematode Community Composition through Ammonium Suppression

**DOI:** 10.1371/journal.pone.0043384

**Published:** 2012-08-31

**Authors:** Cunzheng Wei, Huifen Zheng, Qi Li, Xiaotao Lü, Qiang Yu, Haiyang Zhang, Quansheng Chen, Nianpeng He, Paul Kardol, Wenju Liang, Xingguo Han

**Affiliations:** 1 State Key Laboratory of Vegetation and Environmental Change, Institute of Botany, Chinese Academy of Sciences, Beijing, China; 2 State Key Laboratory of Forest and Soil Ecology, Institute of Applied Ecology, Chinese Academy of Sciences, Shenyang, China; 3 Graduate University of Chinese Academy of Sciences, Beijing, China; 4 Institute of Geographic Sciences and Natural Resources Research, Chinese Academy of Sciences, Beijing, China; 5 Department of Forest Ecology and Management, Swedish University of Agricultural Sciences, Umeå, Sweden; USDA-ARS BARC-W, United States of America

## Abstract

Nitrogen (N) enrichment resulting from anthropogenic activities has greatly changed the composition and functioning of soil communities. Nematodes are one of the most abundant and diverse groups of soil organisms, and they occupy key trophic positions in the soil detritus food web. Nematodes have therefore been proposed as useful indicators for shifts in soil ecosystem functioning under N enrichment. Here, we monitored temporal dynamics of the soil nematode community using a multi-level N addition experiment in an Inner Mongolia grassland. Measurements were made three years after the start of the experiment. We used structural equation modeling (SEM) to explore the mechanisms regulating nematode responses to N enrichment. Across the N enrichment gradient, significant reductions in total nematode abundance, diversity (H' and taxonomic richness), maturity index (MI), and the abundance of root herbivores, fungivores and omnivores-predators were found in August. Root herbivores recovered in September, contributing to the temporal variation of total nematode abundance across the N gradient. Bacterivores showed a hump-shaped relationship with N addition rate, both in August and September. Ammonium concentration was negatively correlated with the abundance of total and herbivorous nematodes in August, but not in September. Ammonium suppression explained 61% of the variation in nematode richness and 43% of the variation in nematode trophic group composition. Ammonium toxicity may occur when herbivorous nematodes feed on root fluid, providing a possible explanation for the negative relationship between herbivorous nematodes and ammonium concentration in August. We found a significantly positive relationship between fungivores and fungal phospholipid fatty acids (PLFA), suggesting bottom-up control of fungivores. No such relationship was found between bacterivorous nematodes and bacterial PLFA. Our findings contribute to the understanding of effects of N enrichment in semiarid grassland on soil nematode trophic groups, and the cascading effects in the detrital soil food web.

## Introduction

Widespread nitrogen (N) enrichment resulting from anthropogenic activities such as N deposition and fertilization has greatly changed ecosystem processes, structure, and functioning [1], [2], [3]. Although low-level N addition generally promotes ecosystem functioning, N saturation has been reported to induce forest dieback, soil acidification and inhibit soil biota [4], [5], [6], [7]. It is therefore important to improve our understanding of the dose-response relationship between N enrichment and soil ecosystem functioning.

Soil nematodes are wide-spread, abundant and highly diverse, both taxonomically and functionally [8], [9], occupying multiple trophic positions in the soil food web, including root herbivores, bacterivores, fungivores, as well as omnivores and predators [10], [11], [12]. Changes in nematode community composition are widely acknowledged as indicative of shifts in environmental conditions [13], [14]. Moreover, soil nematodes can contribute up to 40% of nutrient mineralization by feeding on microbial populations [15], [16]. Thus, understanding the response and underlying mechanisms of soil nematodes to N enrichment will contribute to elucidate potential cascade effects in the soil food web.

Previous studies have documented inhibition of nematodes after N addition [7], [17]. Generally, N addition decreased total nematode abundance and diversity, but responses varied among trophic groups. Reduced numbers of root herbivores, fungivores and omnivores -predators, but increased numbers of some opportunistic bacterivores in response to N addition have been shown for forests [18]–[20], grasslands [17], [18], [21] and croplands [7], [22]. Moreover, responses of soil nematodes to N addition often vary with time after application. For example, in a long-term fertilization experiment in northern China, Liang et al. [7] found that N fertilization initially decreased the abundance of root-feeding nematodes, but increased their abundance the following season.

Soil acidification following N addition has been proposed as one of the important factors inhibiting soil nematode abundance after N addition [21], [23]. In other studies, NO_3_^−^-N and NH_4_^+^-N concentrations were found negatively correlated with root herbivores and fungivores [7], [24], suggesting direct effects of N addition on soil nematodes. Importantly, soil nematodes may not only be influenced by changes in physicochemical soil conditions, but also indirectly by shifts in plant community composition [25]. Altered plant community composition and widespread species loss after N addition has been observed across the world [26], [27]. As nematodes are heterotrophs they are ultimately dependent on autotrophs, such as higher plants, for their resources (i.e. root exudates and litter input) [28]. Plant species diversity and composition likely affect the composition of soil nematodes through the complementarity in resource quality of the component plant species rather than to an increase in total resource quantity [25]. Understanding how N addition affects taxonomic richness and composition of soil nematodes is challenging because of the complex interactions between the direct effects of N addition on soil biota, and indirect effects mediated by altered plant community composition [29].

We used a 4-year multi-level N addition experiment in a typical Inner Mongolia grassland in Northern China to investigate the soil nematode community, the plant community [27], soil biodiversity [21] and key soil physicochemical factors which have been documented to be significantly affected by N addition. We tested the general hypothesis that N enrichment alters soil abiotic properties and plant community composition and species richness which in turn affects nematode community composition and taxon richness. Nematodes were sampled twice during the growing season to address two primary questions. First, how does N enrichment affect temporal fluctuations in nematode abundance and community composition across an N addition gradient? Second, in effects of N enrichment on soil nematode communities, what is the relative importance of direct effects through changes in ammonium and nitrate concentrations, and indirect effects through changes in soil pH and shifts in plant community composition? The causal relationships between components of the plant-soil-nematode system in response to N addition were evaluated using structural equation modeling.

## Materials and Methods

No specific permits were required for the described field studies. No specific permissions were required for these locations/activities. The location is not privately owned or protected in any way and the field studies did not involve endangered or protected species.

### Experimental design

The field experiment was conducted at the Inner Mongolia Grassland Ecosystem Research Station (IMGERS), which is located in the Xilin River Basin, Inner Mongolia Autonomous Region of China (116°42′E, 43°38′N). Topographic relief exhibits little variation, with elevation ranging from 1250 to 1260 m at our experimental site. The region has a semi-arid continental climate with a mean annual temperature of 0.7°C and a mean annual precipitation of 346.1 mm with 60–80% of the precipitation falling during the growing season from May to August. The soil is classified as dark chestnut (Calcic Chernozem according to ISSS Working Group RB, 1998) or loamy sand in terms of texture. Previous experiments have documented that N limitation constrains primary production and regulates plant community composition [27]. The vegetation at the experimental site is classified as typical steppe grassland [30]. The perennial rhizomatous grass *Leymus chinensis* (Trin.) Tsvelev and the perennial bunchgrass *Stipa grandis* P. Smirn are the dominant plant species. Our N manipulation experiment was conducted in *Leymus chinensis* grassland that had been fenced since 1979 to prevent grazing by large animals.

In 2006, 42 8 m×8 m plots were laid out in a randomized block design. Plots were separated by 1-meter walkways. There were seven treatments with six replicates each, including Control (no nutrient addition), and 6 levels of N addition (0, 0.4, 0.8, 1.6, 2.8, 4.0 mol N m^−2^ yr^−1^; N was added as urea). Hereafter, treatments will be referred to as: Control, N_0_, N_0.4_, N_0.8_, N_1.6_, N_2.8_, and N_4.0_. Each plot, except for Control, also received 0.05 mol P m^−2^ (added as KH_2_PO_4_) to ensure that N was the only limiting nutrient. The fertilizer was thoroughly mixed with sand and then applied to the plot surfaces as a single dose at the beginning of the growing season every year from 2006 to 2009 [3], [27], [31].

### Plant sampling and analysis

The aboveground vegetation was sampled each year in mid-August by clipping all plants at the soil surface using a 0.5 m×1 m quadrat randomly placed in each plot with the restriction of no spatial overlap of quadrats across years. All living vascular plants were sorted to species, and all plant materials, including litter and standing dead biomass, were oven-dried at 65°C for 48 h and then weighted. The dry mass of all living plants per quadrat averaged over the six replicates for each treatment was used to estimate aboveground biomass. We classified the plants into five functional groups (PFGs) based on life forms, including perennial rhizome grass (PR), perennial bunchgrasses (PB), perennial forbs (PF), shrubs and semi-shrubs (SS), and annuals (AS), as in Bai et al. [30]. Two soil cores with a diameter of 6.5 cm were collected at 0–15 cm depth to determine root biomass from each plot after plant biomass harvest. Root samples were immediately placed in a cooler and transported to the laboratory. In the laboratory, root samples were soaked in deionized water and cleaned from soil residuals using a 0.5-mm sieve. Root biomass was then dried and weighed as described above.

### Soil sampling and analysis

In 2009, three years after the start of the experiment, from each plot soil samples were first collected on June 10 (when the rain season started and urea dissolved), and then on August 18 and September 19. From each plot, three soil cores (3 cm diameter, 0–15 cm depth) were collected. Per plot, the three soil cores were bulked comprising one soil sample per plot. Bulked samples were placed in a plastic bag and then immediately transported to the laboratory and stored at 4°C. Soil moisture (SM) was determined as mass loss after drying the soils at 105°C for 24 h. Part of the air-dried and sieved samples was prepared for measurement of pH and total organic carbon. For measurement of soil inorganic N, soil samples were extracted using 2 mol L^−1^ KCl. The concentrations of nitrate N (NO_3_^−^-N) and ammonium N (NH_4_^+^-N) in the filtrates were determined using a flow injection auto analyzer (FIAstar 5000 Analyzer; Foss Tecator, Hillerød, Denmark). The concentrations of soil inorganic N were calculated based on dry soil weight.

### Extraction and identification of soil nematodes

Soil nematodes were extracted from 100 g subsamples collected in August and September 2009 using a modified cotton-wool filter method [32]. After counting the total number of nematodes, the first 100 individuals encountered were identified to genus level using an inverted compound microscope. If the total number of nematodes was less than 100, all nematodes were identified. Nematode populations are expressed as number of nematodes per 100 g dry soil. The nematodes were allocated to the following trophic groups: root herbivores, bacterivores, fungivores, and omnivores–predators [10].

### Data analysis

August and September physicochemical soil properties used in the SEM model and linear regression analyses (see below) were calculated as the mean of July and August, and the mean of August and September, respectively.

The following nematode community indices were calculated: 1) Nematode taxon richness (S), i.e., the number of genera identified [25]; 2) The Shannon diversity index H' = −∑p_i_(lnp_i_), where p_i_ is the proportion of individuals belonging to the i^th^ taxon relative to the total number of individuals in the sample; and 3) The maturity index MI = Σv(i)f(i), where v(i) is the c-p value of taxon i indicating their r and K strategies [33], and f(i) is the frequency of taxon i in a sample. Nematode abundance and ecological indices were ln(x+1)-transformed prior to statistical analysis. The main and interactive effects of N addition and sampling time (August, September) on soil nematode abundance and ecological indices were determined using two-way analysis of variance (ANOVA). Polynomial and exponential decay regressions were used to test relationships of nematode abundance and diversity indices with N addition rate; results from the best-fitting regression models were presented.

Structural equation modeling (SEM) was used to gain a mechanistic understanding of how soil properties and altered plant community composition mediate effects of N enrichment on soil nematode communities. SEM is based on a simultaneous solution procedure, where the residual effects of predictors are estimated (partial regressions) once common causes from inter-correlations have been statistically controlled for [34]–[36]. Prior to the SEM procedure we reduced the number of variables for plant and nematode community composition through principal component analyses (PCA) [35]. Plant functional group biomass and abundance of nematode trophic groups were used as raw data for the PCAs. The first principal components (PC) were used in the subsequent SEM analysis to represent nematode community composition (PC1 explained 76.7% of the variation, Fig. S1), and plant community composition (PC1 explained 87.5% of the variation, Fig. S2). We started the SEM procedure with the specification of a conceptual model of hypothetical relationships based on *a priori* and theoretical knowledge (Fig. S3). In this model, we assumed that grazing alters soil abiotic properties and plant community composition and species richness which in turn affect nematode community composition and taxon richness. In the SEM analysis we compared the model-implied variance-covariance matrix against observed variance-covariance matric. Data were fitted to the models using the maximum likelihood estimation method. The *χ^2^* goodness-of-fit statistic and its associated P value, Root Mean Square Error of Approximation (RMSEA) and Akaike Information Criterion (AIC) were used to judge the model fit to the data. A large P value and a low RMSEA value indicates that the covariance structure of the data does not differ significantly from what would be expected based on the model.

All univariate analyses were performed using SPSS 17.0 (SPSS, Chicago, IL). PCA was performed using Program CANOCO Version 4.5 (Plant Research International, Wageningen, The Netherlands). SEM analyses were performed using AMOS 7.0 (Amos Development, Spring House, Pennsylvania, USA).

## Results

### Soil N and plant responses to N addition

**Figure 1:**
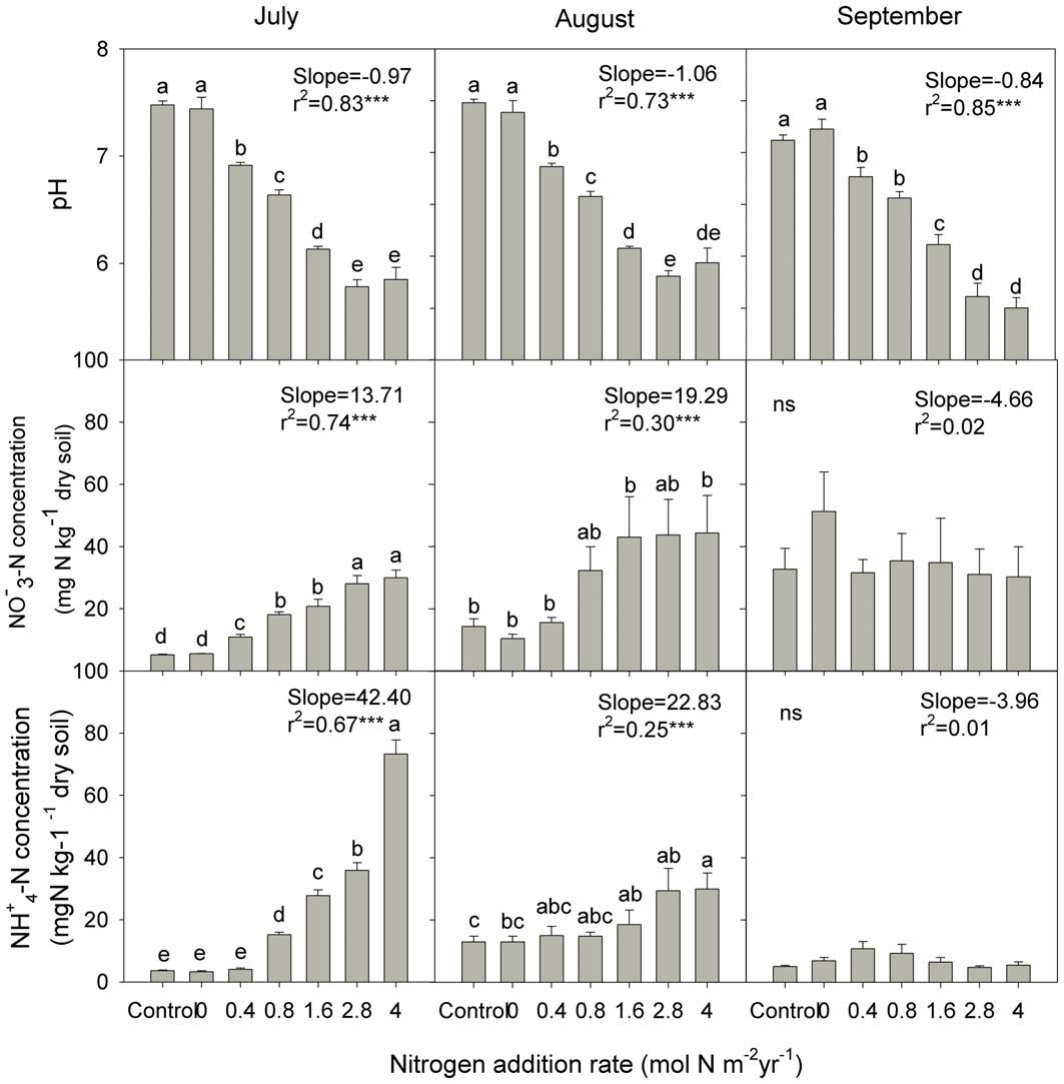
Soil ammonium (NH_4_^+^), nitrate (NO_3_^−^) concentration, and pH for Control and N addition treatments in July, August and September 2009. Data are mean ± S.E. (N = 6). Regression parameters were estimated using log-linear models with N treatment as a continuous predictor. Significant regressions are reported as *P*<0.05*, *P*<0.01**, *P*<0.001***.

Nitrogen addition greatly increased nitrate and ammonium concentrations in July (Fig. 1). However, effects of N addition on nitrate and ammonium concentrations decreased during the growing season. In August, nitrate and ammonium concentrations were only somewhat increased at the highest N addition levels. In September, nitrate and ammonium concentrations did not significantly differ between Control and N addition treatments. Across the growing season, pH was significantly decreased with increasing levels of N addition (Fig. 1).

Nitrogen addition significantly reduced plant species richness from 7 at N_0_ to 4 at N_4_ plots (F_6,35_ = 4.95, *P*<0.001; Table S1). Plant shoot and root biomass were not significantly affected by N addition (F_6,35_ = 1.34, *P* = 0.27; F_6,35_ = 1.57, *P* = 0.19 respectively; Table S1). Plant functional groups varied in their response to N addition. For example, shoot biomass of perennial bunchgrasses (PB) increased at low levels of N addition, but decreased at high levels of N addition, with a threshold at 0.4 mol N m^−2^ y^−1^ (F_6,35_ = 2.314, *P* = 0.074; Table S1). In contrast, shoot biomass of perennial rhizome grasses (PR) showed the exact opposite pattern, although the effects of N additions were not statistically significant (F_6,35_ = 1.633, *P* = 0.167; Table S1). No significant responses to N addition were found for perennial forbs (PF) and for (semi-)shrubs (SS) (F_6,35_ = 0.954, *P* = 0.470; F_6,35_ = 1.048, *P* = 0.412, respectively; Table S1).

### Nematode responses to N addition

**Figure 2:**
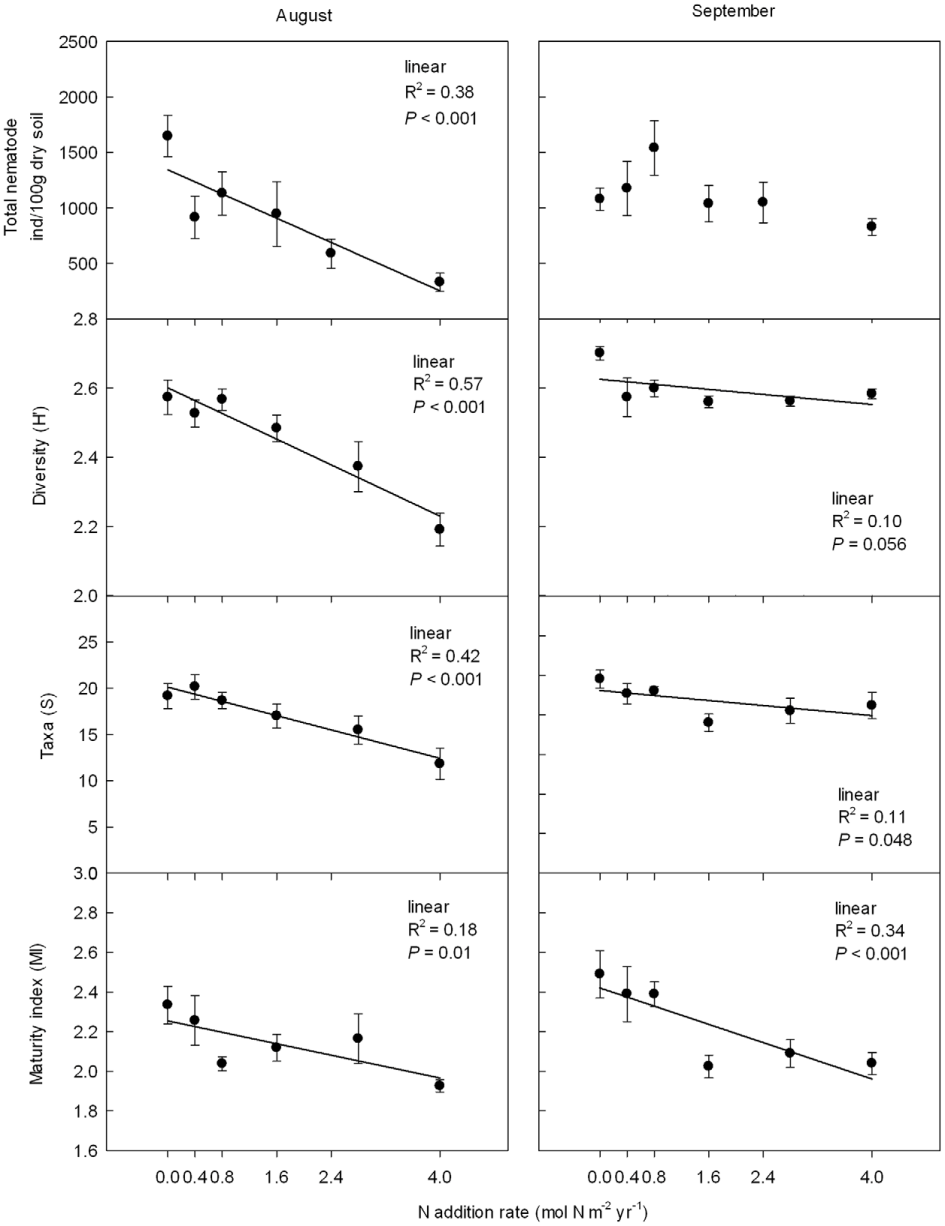
Response of soil nematode abundance, diversity (H'), taxon richness (S), and maturity index (MI) to N addition in August and September 2009. Data are means ± S.E. Linear regressions best fitted the data; regression coefficients are reported if *P*<0.05.

Thirty nine and fifty nematode genera were identified in August and September respectively (Table S3, Table S4). Effects of N addition on nematode community diversity (H') strongly changed during the growing season, as indicated by the significant treatment×time interaction effect (Table 1). Nematode abundance, diversity (H' and S) and MI all linearly decreased with the level of N addition in August (Fig. 2). In contrast, no effect of N addition on nematode abundance was found in September (Table 1; Fig. 2). Nematode community diversity (H') and taxon richness (S) both decreased with N addition (*P* = 0.054 and 0.048, respectively) in September, but the effects were much weaker than in August (Fig. 2). Both in August and September the MI decreased linearly with N addition, while on average the MI was higher in September than in August (Fig. 2).

**Table 1:** Results from two-way analysis of variance for total and nematode feeding group abundance and nematode ecological indices using N treatment (N), sampling time (T) and their interaction as fixed factors.

Dependent variable	Error df	Treatment (6df)	Time (1df)	N*T (6df)
Total nematode abundance	84	**4.15 (0.001)**	3.50 (0.066)	1.76 (0.119)
Nematode taxa (S)	84	**6.25 (<0.001)**	**43.30 (<0.001)**	1.61 (0.157)
Diversity (H')	84	**10.06 (<0.001)**	**62.19 (<0.001)**	**5.87 (<0.001)**
Maturity index (MI)	84	**6.96 (<0.001)**	**6.16 (0.015)**	1.64 (0.149)
Root herbivores	84	**2.25 (0.048)**	**4.31 (0.042)**	**2.77 (0.018)**
Fungivores	84	**8.63 (<0.001**)	**3.74 (0.057)**	1.74 (0.125)
Bacterivores	84	1.42 (0.218)	**54.36(<0.001)**	0.30 (0.936)
Omnivores-predators	84	**4.895 (0.001)**	0.16 (0.687)	1.98 (0.080)

Shown are F-values with significance levels in parentheses. Significant effects (*P*<0.05) are indicated in bold and marginally significant effects are indicated in italic.

**Figure 3:**
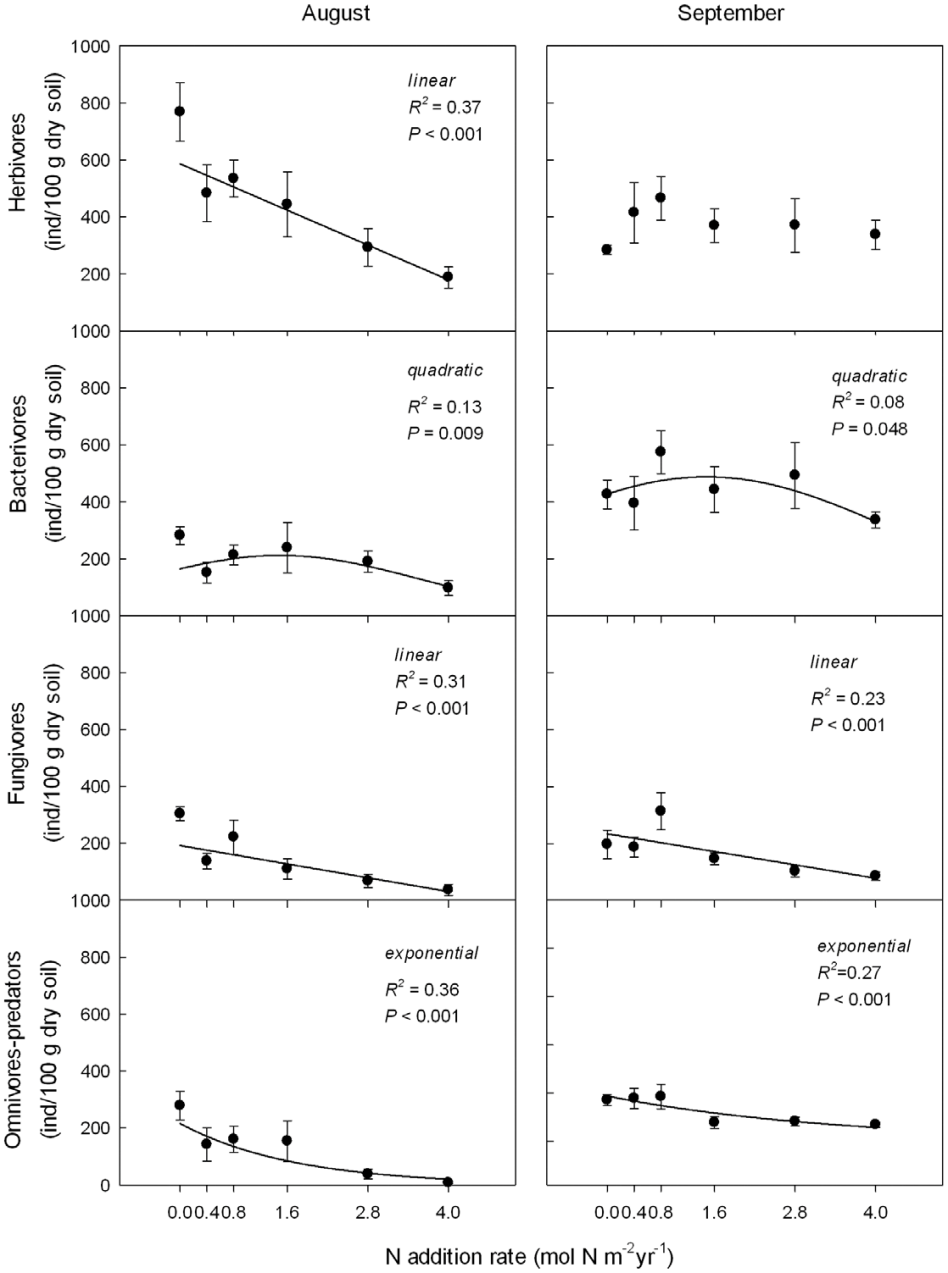
Response of nematode tropic groups to N addition in August and September 2009. Data are means ± S.E.. R^2^ values were determined using polynomial and exponential decay regressions. Regression coefficients of the best-fitting models are reported if *P*<0.05.

Nematode trophic groups showed a diverse response to the N addition treatments. N addition negatively affected the abundance of fungivores; their numbers linearly decreased with N addition. The abundance of fungivores was on average higher in August than in September (Table 1; Fig. 3). N addition also negatively affected the abundance of omnivores-predators, but the effect of N was stronger in August than in September. Across treatments, omnivores-predators were on average more abundant in September than in August (Table 1; Fig. 3). Root herbivores were strongly negatively affected by N addition in August, but not in September, resulting in a significant treatment×time interaction (Table 1). Analysis of variance did not reveal an overall effect of N addition on the abundance of bacterivores (Table 1; Fig. 3). Regression models showed that bacterivorous nematodes had a hump-shaped relationship with the level of N addition, but without a critical threshold (Fig. 3).

### Direct and indirect effects of N addition on soil nematode communities

**Figure 4:**
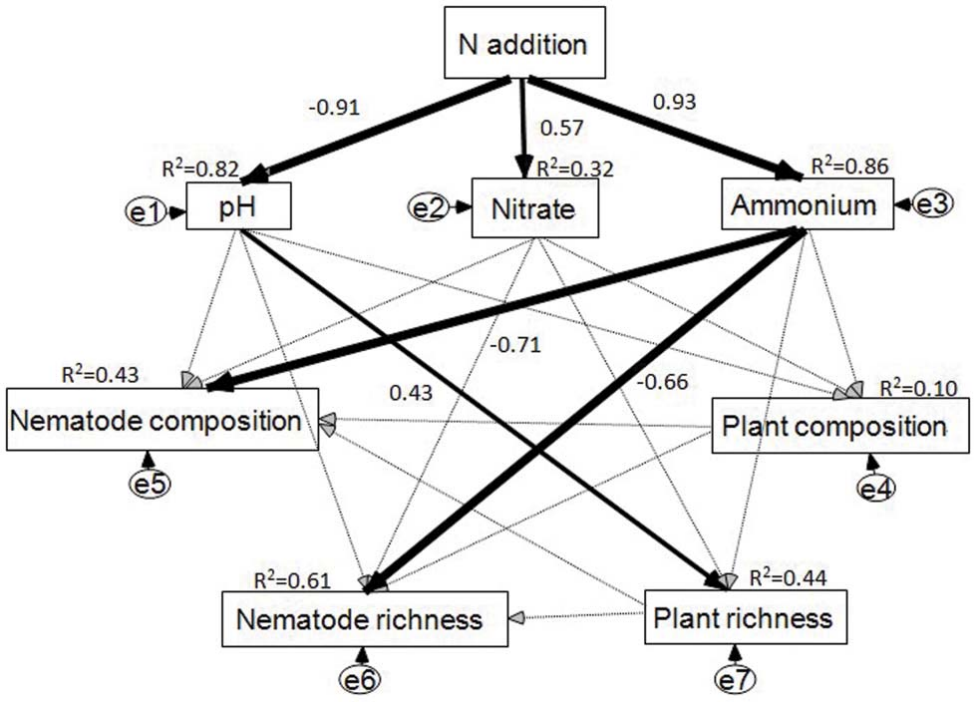
Structural equation model of N addition on soil physicochemical factors, plant community composition and richness, nematode community composition and richness (August data was used in SEM; ammonium, nitrate concentration was calculated as the average of July and August). The model fit the data well: *χ^2^_9_* = 8.286, *P* = 0.507, AIC = 62.268, RMSEA<0.001. Numbers at arrows are standardized path coefficients (equivalent to correlation coefficients). Width of the arrows indicates the strength of the causal influences: black arrows indicate significant standardized path coefficients (*P*<0.05). Circles indicate error terms (e1–e7). Percentages close to endogenous variables indicate the variance explained by the model (R^2^).

**Figure 5:**
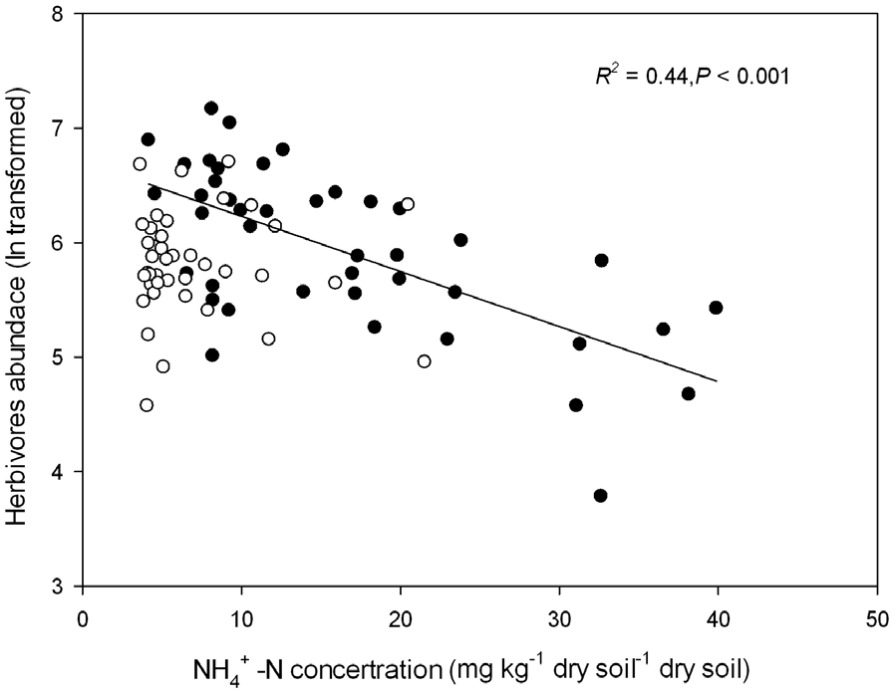
Relationships between soil ammonium (NH_4_^+^) concentration and abundance of herbivorous nematodes in August and September 2009 (close circles: August, open circles: September; ammonium concentration was calculated as mean value of July and August, August and September respectively). In August, abundance of herbivorous nematodes correlated negatively with NH_4_^+^-concentration.

The final SEM model adequately fitted the data describing effects of N addition on the plant-soil-nematode system (*χ^2^_9_* = 8.286, *P* = 0.507, AIC = 62.268, RMSEA<0.001; Standardized path coefficients are given in Fig. 4). Nitrogen addition explained 82%, 32% and 86% of the variation in soil pH, nitrate concentration, and ammonium concentration, respectively. Ammonium concentration (Fig. 4) had a negative relationship with nematode composition and richness (*P*<0.01, Table S2). Ammonium suppression explained 61% of the variation in nematode richness and 43% of the variation in nematode community composition. However, we did not find any significant effects of altered plant community composition on nematode richness and community composition. Also, effects of soil pH and nitrate concentration on nematode richness and community composition were not significant (Fig. 4; Table S2). A further regression model showed that ammonium concentration had a strong negative relationship with abundance of herbivorouos nematodes in August, but not in September (Fig. 5).

## Discussion

### Ammonium suppression of herbivorous nematodes after N addition

We observed a decrease in total soil nematode abundance and diversity after N addition, which was consistent with previous findings in a nearby NH_4_NO_3_ addition experiment [21]. Herbivorous nematodes were the primary contributors to the temporal dynamics of nematode community structure across the N addition gradient (Fig. 2, Fig. 3). Persson et al. (1980) found a strong reduction of the obligatory root-feeder *Geocenamus arcticus* in responses to N fertilization in a Scots Pine forest, even though root production increased [37]. In our experiment, plant root biomass did not change across the N gradient (Table S1). So, parasite-host relationships could not explain the decrease of herbivorous nematode abundance following N addition.

Structural equation modeling showed that ammonium concentration alone significantly contributed to the shift in nematode community composition and diversity (Fig. 4). Further analysis showed a negative relationship between ammonium concentration and herbivorous nematodes along the N addition gradient in August (Fig. 5). Ammonium suppression by fertilizers of plant parasitic nematodes has been documented before and ammonium has been used as a nematicide to control root-knot nematodes [24], [38]. In a recent paper, Oka et al. (2007) found that ammonium and ammonia toxicity after soil solarization could be used to control root-knot nematodes in organic farming systems [39]. Cumulative ammonium concentrations were observed initially after urea addition (Fig. 1). Ammonium can be taken up by plants and be nitrified by nitrobacteria under certain conditions [40]. Temporal depletion of ammonium was also observed in August and September (Fig. 4). Alleviation of ammonium suppression could explain the temporal recovery of abundance of herbivorous nematodes, which also has been found in long-term N fertilization of maize crops [7].

### Responses of non-herbivorous nematodes to N addition

Ammonium has been documented toxic to a wide range of organisms [41]. However, ammonium toxicity could not well explain the responses of microbial-feeding and omnivorous-predatory nematodes to N addition in our experiment. These contrasting responses might be due to their disparate feeding habits. Plants can take up ammonium directly from soil and accumulate it in their roots, stems and leaves [42]. Herbivorous nematodes parasitize plant roots by puncturing plant cells with their stylet, and ingestion of ammonium-rich fluid might cause ammonium toxicity [10]. In contrast, other nematode trophic groups either feed on microorganisms or other microfauna in which case ammonium might already have been transformed to non-toxic compounds.

Bottom up control of fungivores by fungi was evident from the significant negative relationship between fungal PLFA and abundance of fungivores (Fig. S4). Cascading effects are suggested to be a universal mechanism regulating soil food web structure [29], [43] and our experiment provides further evidence. However, bacterivores did not show a relationship with bacterial PLFA, which suggest other controlling mechanisms, such as top down controls by their predaceous soil organisms [43]–[45].

### Indirect effects of altered plant community composition on soil nematodes after N addition

In contrast to Bardgett et al. [46] who showed that in a short-term pot experiment abundance and activity of soil organisms were regulated more by plant species traits than by the direct effects of N addition, we did not find evidence for indirect effects of N addition on soil nematode communities through shifts in plant community composition (Fig. 3). Bottom-up effects from the plant community to soil fauna have long been documented, as plants fix carbon, which is often a limiting resource for soil biota [47]. However, nitrogen saturation resulting from high levels of N addition can strongly deteriorate soil properties (e.g., acidification and ammonium toxicity as shown in our experiment) [48], which may outweigh plant effects on soil biota and soil processes. Considering the high levels of N addition in our experiment, soil properties could therefore probably be expected to be more important than shifts in plant community composition in regulating soil nematode communities (Wei et al. unpublished). Similarly, previous microcosm studies have shown that direct effects of N enrichment on litter decomposition and ecosystem functioning can be stronger than indirect effects through changes in the plant community [49], [50]. However, it should be noted that N-induced shifts in plant community composition may simply take longer to exert effects on the soil ecosystem than direct effect of N addition; hence, longer-term studies are needed to further evaluate the relative importance of direct and indirect effects of N addition.

## Supporting Information

Figure S1 Species-sample bi-plot of principal component analysis (PCA) of soil nematode community composition. Bac = bacterivores abundance, Fun = fungivores abundance, Her = root herbivores abundance, OP = omnivores-predators abundance. Percentages along the axes correspond to the amount of explained variability in functional group composition.
(DOCX)

Figure S2 Species-sample bi-plot of principal component analysis (PCA) of plant community composition. TolBio = Total aboveground biomass, PR = perennial rhizome grass, PB = perennial bunchgrasses, PF = perennial forbs, SS = shrubs and semi-shrubs. Percentages along the axes correspond to the amount of explained variability in functional group composition.
(DOCX)

Figure S3 Conceptual model of hypothetical interaction pathways in the studied plant-soil-nematode system.
(DOCX)

Figure S4 Relationships between bacterial phospholipid fatty acids (PLFA) and abundance of bacterivorous nematodes, fungal PLFA and fungivorous nematodes. Data are ln transformed.
(DOCX)

Table S1 Plant richness, and shoot, root, and functional group shoot biomass (g m^−2^) for Control and N addition treatments. Values shown are mean ± s.d. (N = 6). Significant differences among treatments are indicated by different letter superscripts (*P*<0.05). PR = perennial rhizome grasses, PB = perennial bunch grasses, PF = perennial forbs, SS = shrubs and semi-shrubs. N_0_ = 0 mol N m^−2^ y^−1^, N_0.4_ = 0.4 mol N m^−2^ y^−1^, N_0.8_ = 0.8 mol N m^−2^ y^−1^, N_1.6_ = 1.6 mol N m^−2^ y^−1^, N_2.8_ = 2.8 mol N m^−2^ y^−1^, N_4_ = 4.0 mol N m^−2^ y^−1^.
(DOCX)

Table S2 Results of structural equation modeling of N addition effects on the plant-soil-nematode system as illustrated in Fig. 3 (main text). Given are the unstandardized path coefficients (estimates), standard error of regression weight (S.E.), the critical value for regression weight (C.R.; z = estimate/S.E.) and the level of significance for regression weight (p). For more information on exogenous and endogenous variables as well as model fit, see main text (*** *P*<0.001).
(DOCX)

Table S3 Relative abundance of nematode genera (%) for Control and N treatments in August 2009. Data are mean values (N = 6). H = herbivores, Ba = bacterivores, Fu = fungivores, Om = omnivores, Ca = carnivores.
(DOCX)

Table S4 Relative abundance of nematode genera (%) for Control and N addition treatments in September 2009. Data are mean values (N = 6). H = herbivores, Ba = bacterivores, Fu = fungivores, Om = omnivores, Ca = carnivores.
(DOCX)
