# Supplementary material for: Nitrogen Addition Regulates Soil Nematode Community Composition through Ammonium Suppression
Source: PLoS One. 2012 Aug 31;7(8):e43384. doi: 10.1371/journal.pone.0043384 (PMC3432042; doi:10.1371/journal.pone.0043384)
Supplement: Figure S1 — Species-sample bi-plot of principal component analysis (PCA) of soil nematode community composition. Bac = bacterivores abundance, Fun = fungivores abundance, Her = root herbivores abundance, OP = omnivores-predators abundance. Percentages along the axes correspond to the amount of explained variability in functional group composition. (DOCX) [file pone.0043384.s001.docx]

Fig S1. Species-sample bi-plot of principal component analysis (PCA) of soil nematode community composition. Bac = bacterivores abundance, Fun = fungivores abundance, Her = root herbivores abundance, OP = omnivores-predators abundance. Percentages along the axes correspond to the amount of explained variability in functional group composition.

PC2 (12.8%)

-0.2

1.0

-0.4

0.6

**Bac**

**Fun**

**Her**

**OP**

PC1 (76.7%)
