# Supplementary material for: Nitrogen Addition Regulates Soil Nematode Community Composition through Ammonium Suppression
Source: PLoS One. 2012 Aug 31;7(8):e43384. doi: 10.1371/journal.pone.0043384 (PMC3432042; doi:10.1371/journal.pone.0043384)
Supplement: Figure S2 — Species-sample bi-plot of principal component analysis (PCA) of plant community composition. TolBio = Total aboveground biomass, PR = perennial rhizome grass, PB = perennial bunchgrasses, PF = perennial forbs, SS = shrubs and semi-shrubs. Percentages along the axes correspond to the amount of explained variability in functional group composition. (DOCX) [file pone.0043384.s002.docx]

Fig S2. Species-sample bi-plot of principal component analysis (PCA) of plant community composition. TolBio = Total aboveground biomass, PR = perennial rhizome grass, PB = perennial bunchgrasses, PF = perennial forbs, SS = shrubs and semi-shrubs. Percentages along the axes correspond to the amount of explained variability in functional group composition.

-1.0

1.0

**PR**

**PB**

**PF**

**SS**

PC1 (87.5%)

PC2 (11.2%)

1.0

-1.0
