# Supplementary material for: Nitrogen Addition Regulates Soil Nematode Community Composition through Ammonium Suppression
Source: PLoS One. 2012 Aug 31;7(8):e43384. doi: 10.1371/journal.pone.0043384 (PMC3432042; doi:10.1371/journal.pone.0043384)
Supplement: Figure S3 — Conceptual model of hypothetical interaction pathways in the studied plant-soil-nematode system. (DOCX) [file pone.0043384.s003.docx]

Fig S3. Conceptual model of hypothetical interaction pathways in the studied plant-soil-nematode system.

N addition

pH

Nitrate

Ammonium

Nematode composition

Nematode richness

Plant richness

Plant composition

e1

e2

e3

e4

e5

e6

e7
