# Supplementary material for: Nitrogen Addition Regulates Soil Nematode Community Composition through Ammonium Suppression
Source: PLoS One. 2012 Aug 31;7(8):e43384. doi: 10.1371/journal.pone.0043384 (PMC3432042; doi:10.1371/journal.pone.0043384)

Fig S4. Relationships between bacterial phospholipid fatty acids (PLFA) and abundance of bacterivorous nematodes, fungal PLFA and fungivorous nematodes. Data are ln transformed.


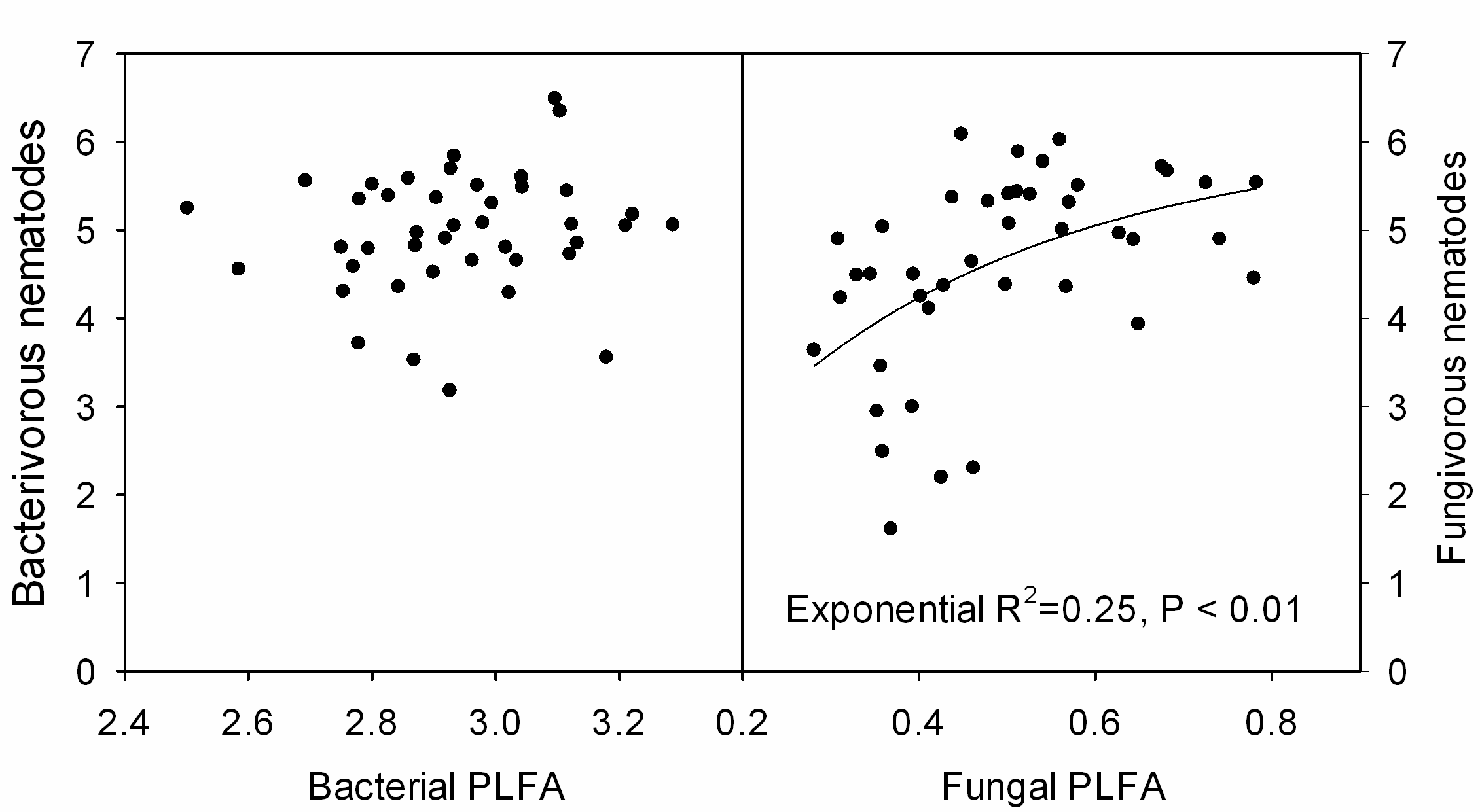

Supplement: Figure S4 — Relationships between bacterial phospholipid fatty acids (PLFA) and abundance of bacterivorous nematodes, fungal PLFA and fungivorous nematodes. Data are ln transformed. (DOCX) [file pone.0043384.s004.docx]
