# Supplementary material for: Nitrogen Addition Regulates Soil Nematode Community Composition through Ammonium Suppression
Source: PLoS One. 2012 Aug 31;7(8):e43384. doi: 10.1371/journal.pone.0043384 (PMC3432042; doi:10.1371/journal.pone.0043384)
Supplement: Table S1 — Plant richness, and shoot, root, and functional group shoot biomass (g m−2) for Control and N addition treatments. Values shown are mean ± s.d. (N = 6). Significant differences among treatments are indicated by different letter superscripts (P<0.05). PR = perennial rhizome grasses, PB = perennial bunch grasses, PF = perennial forbs, SS = shrubs and semi-shrubs. N0 = 0 mol N m−2 y−1, N0.4 = 0.4 mol N m−2 y−1, N0.8 = 0.8 mol N m−2 y−1, N1.6 = 1.6 mol N m−2 y−1, N2.8 = 2.8 mol N m−2 y−1, N4 = 4.0 mol N m−2 y−1. (DOCX) [file pone.0043384.s005.docx]

Table S1. Plant richness, and shoot, root, and functional group shoot biomass (g m^-2^) for Control and N addition treatments. Values shown are mean ± s.d. (N = 6). Significant differences among treatments are indicated by different letter superscripts (*P* < 0.05). PR = perennial rhizome grasses, PB = perennial bunch grasses, PF = perennial forbs, SS = shrubs and semi-shrubs. N_0_ = 0 mol N m^-2^ y^-1^, N_0.4_ = 0.4 mol N m^-2^ y^-1^, N_0.8_= 0.8 mol N m^-2^ y^-1^, N_1.6_ = 1.6 mol N m^-2^ y^-1^, N_2.8_ =2.8 mol N m^-2^ y^-1^, N_4_ =4.0 mol N m^-2^ y^-1^.

|  | Control | N0 | N0.4 | N0.8 | N1.6 | N2.8 | N4 |
| --- | --- | --- | --- | --- | --- | --- | --- |
| Richness | 6 (±1)abc | 7 (±2)a | 6 (±1)ab | 5 (±0)bcd | 5 (±1)bcd | 4 (±1)d | 4 (±1)cd |
| Shoot biomass | 81.07 (±6.30) | 81.37 (±12.24) | 74.83 (±25.67) | 71.77 (±11.08) | 72.20 (±20.95) | 63.37 (±19.82) | 56.37 (±28.01) |
| Root biomass | 326.9 (±74.0) | 498.4 (±330.8) | 334.3 (±210.5) | 323.5 (±93.4) | 411.0 (108.1) | 459.9 (±158.7) | 396.7 (±248.1) |
| PR | 45.4 (±34.4)^ab^ | 34.8 (±16.3)^ab^ | 10.9 (±6.8)^a^ | 16.3 (±19.1)^ab^ | 34.6 (31.5)^ab^ | 41.2 (±20.9)^b^ | 35.1 (±30.0)^b^ |
| PB | 29.3 (±34.8)^a^ | 38.9 (±19.8)^ab^ | 59.2 (±18.78)^b^ | 50.4 (±20.8)ab | 33.7 (±11.6)ab | 21.1 (±12.6)^ab^ | 18.5 (±31.0)^ab^ |
| PF | 4.72 (±2.76) | 6.65 (±4.48) | 4.04 (±5.93) | 4.69 (±1.71) | 3.89 (±5.02) | 1.07 (±1.70) | 2.81 (±6.22) |
| SS | 1.56 (±3.29) | 1.07 (±1.61) | 0.65 (±0.95) | 0.35 (±0.83) | 0 | 0 | 0 |
