# Supplementary material for: Nitrogen Addition Regulates Soil Nematode Community Composition through Ammonium Suppression
Source: PLoS One. 2012 Aug 31;7(8):e43384. doi: 10.1371/journal.pone.0043384 (PMC3432042; doi:10.1371/journal.pone.0043384)
Supplement: Table S2 — Results of structural equation modeling of N addition effects on the plant-soil-nematode system as illustrated in Fig. 3 (main text). Given are the unstandardized path coefficients (estimates), standard error of regression weight (S.E.), the critical value for regression weight (C.R.; z = estimate/S.E.) and the level of significance for regression weight (p). For more information on exogenous and endogenous variables as well as model fit, see main text (*** P<0.001). (DOCX) [file pone.0043384.s006.docx]

Table S2. Results of structural equation modeling of N addition effects on the plant-soil-nematode system as illustrated in Fig. 3 (main text). Given are the unstandardized path coefficients (estimates), standard error of regression weight (S.E.), the critical value for regression weight (C.R.; z=estimate/ S.E.) and the level of significance for regression weight (p). For more information on exogenous and endogenous variables as well as model fit, see main text (*** *P* < 0.001).

|  |  |  | Estimate | S.E. | C.R. | P |
| --- | --- | --- | --- | --- | --- | --- |
| Ammonium | <--- | N addition | 6.399 | .401 | 15.944 | *** |
| Nitrate | <--- | N addition | 5.626 | 1.279 | 4.398 | *** |
| pH | <--- | N addition | -.451 | .033 | -13.769 | *** |
| Plant richness | <--- | pH | .876 | .444 | 1.975 | .048 |
| Plant richness | <--- | Nitrate | .017 | .014 | 1.194 | .233 |
| Plant richness | <--- | Ammonium | -.049 | .032 | -1.532 | .125 |
| Plant composition | <--- | Nitrate | -.021 | .012 | -1.660 | .097 |
| Plant composition | <--- | pH | .243 | .390 | .622 | .534 |
| Plant composition | <--- | Ammonium | .046 | .028 | 1.613 | .107 |
| Nematode composition | <--- | pH | -.008 | .328 | -.025 | .980 |
| Nematode composition | <--- | Nitrate | .012 | .010 | 1.193 | .233 |
| Nematode richness | <--- | Plant richness | -.347 | .313 | -1.107 | .268 |
| Nematode composition | <--- | Plant composition | .122 | .125 | .978 | .328 |
| Nematode richness | <--- | pH | 1.487 | .935 | 1.590 | .112 |
| Nematode richness | <--- | Nitrate | .044 | .030 | 1.485 | .138 |
| Nematode richness | <--- | Plant composition | -.261 | .356 | -.733 | .463 |
| Nematode composition | <--- | Plant richness | .037 | .110 | .338 | .735 |
| Nematode richness | <--- | Ammonium | -.231 | .069 | -3.371 | *** |
| Nematode composition | <--- | Ammonium | -.072 | .024 | -3.011 | .003 |
