# Supplementary material for: Nitrogen Addition Regulates Soil Nematode Community Composition through Ammonium Suppression
Source: PLoS One. 2012 Aug 31;7(8):e43384. doi: 10.1371/journal.pone.0043384 (PMC3432042; doi:10.1371/journal.pone.0043384)
Supplement: Table S3 — Relative abundance of nematode genera (%) for Control and N treatments in August 2009. Data are mean values (N = 6). H = herbivores, Ba = bacterivores, Fu = fungivores, Om = omnivores, Ca = carnivores. (DOCX) [file pone.0043384.s007.docx]

Table S3. Relative abundance of nematode genera (%) for Control and N treatments in August 2009. Data are mean values (N = 6). H = herbivores, Ba = bacterivores, Fu = fungivores, Om = omnivores, Ca = carnivores.

| Genus | Guild | N addition level | | | | | | |
| --- | --- | --- | --- | --- | --- | --- | --- | --- |
|  |  | Control | N_0_ | N_0.4_ | N_0.8_ | N_1.6_ | N_2.4_ | N_4.0_ |
| *Geocenamus* | H2 | 10.3 | 10.0 | 17.7 | 11.2 | 6.9 | 5.0 | 10.8 |
| *Amplimerlinius* | H2 | 1.2 | 3.1 | 1.0 | 1.3 | 2.0 | 0.4 | 0.2 |
| *Nagelus* | H2 | 4.8 | 6.1 | 4.8 | 4.9 | 3.4 | 3.5 | 11.3 |
| *Paratylenchus* | H2 | 0.9 | 0.2 | 0.9 | 0.8 | 0.8 | 0.8 | 0.0 |
| *Helicotylenchus* | H3 | 22.7 | 16.7 | 18.6 | 21.4 | 28.4 | 31.0 | 24.8 |
| *Pararotylenchus* | H3 | 5.6 | 5.8 | 7.5 | 7.9 | 6.2 | 6.0 | 7.9 |
| *Rotylenchus* | H3 | 2.3 | 1.1 | 1.2 | 1.3 | 2.1 | 2.1 | 2.7 |
| *Hemicriconemoides* | H3 | 0.3 | 0.2 | 0.0 | 0.3 | 0.5 | 0.2 | 0.0 |
| *Pratylenchus* | H3 | 0.5 | 0.2 | 0.3 | 0.6 | 0.2 | 0.2 | 0.7 |
| *Longidorus* | H5 | 0.3 | 0.0 | 0.1 | 0.0 | 0.0 | 0.2 | 0.0 |
| *Panagrolaimus* | Ba1 | 0.5 | 0.0 | 0.3 | 0.2 | 0.2 | 0.8 | 0.0 |
| *Cephalobus* | Ba2 | 3.4 | 2.9 | 2.1 | 5.5 | 5.7 | 12.3 | 13.3 |
| *Cervidellus* | Ba2 | 5.6 | 5.9 | 7.5 | 6.3 | 8.0 | 4.8 | 6.4 |
| *Acrobeles* | Ba2 | 4.4 | 3.1 | 1.6 | 3.3 | 2.4 | 1.0 | 0.5 |
| *Chiloplacus* | Ba2 | 0.2 | 0.0 | 0.0 | 0.2 | 0.0 | 1.0 | 2.0 |
| *Hetetorocephalobus* | Ba2 | 0.2 | 0.6 | 1.8 | 0.6 | 0.2 | 0.4 | 0.5 |
| *Acrobeloides* | Ba2 | 0.8 | 1.0 | 1.2 | 0.9 | 4.2 | 11.6 | 5.4 |
| *Acrobelophis* | Ba2 | 0.3 | 1.3 | 0.0 | 0.0 | 0.0 | 0.0 | 0.0 |
| *Stegella* | Ba2 | 1.2 | 0.5 | 1.3 | 1.3 | 2.3 | 1.2 | 1.2 |
| *Paramphidelus* | Ba4 | 1.1 | 1.0 | 0.7 | 0.8 | 0.7 | 0.2 | 0.5 |
| *Boleodorus* | Fu2 | 0.2 | 0.0 | 0.0 | 0.2 | 0.0 | 0.2 | 0.0 |
| *Coslenchus* | Fu2 | 5.2 | 3.2 | 1.3 | 1.4 | 3.8 | 1.9 | 2.0 |
| *Malenchus* | Fu2 | 1.9 | 2.7 | 2.5 | 1.1 | 1.0 | 2.3 | 0.2 |
| *Tylenchus* | Fu2 | 1.7 | 2.9 | 0.9 | 1.6 | 0.8 | 0.2 | 0.2 |
| *Filenchus* | Fu2 | 3.3 | 4.6 | 6.1 | 5.7 | 3.3 | 2.1 | 1.0 |
| *Paraphelenchus* | Fu2 | 2.0 | 1.8 | 1.0 | 4.9 | 2.3 | 4.2 | 5.7 |
| *Funaria* | Fu4 | 3.6 | 3.7 | 5.6 | 3.1 | 1.8 | 0.4 | 0.0 |
| *Tylencholaimus* | Fu4 | 0.8 | 1.0 | 0.7 | 0.3 | 0.0 | 0.0 | 0.0 |
| *Thonus* | Om4 | 0.5 | 1.0 | 1.3 | 1.1 | 0.2 | 0.2 | 0.0 |
| *Microdorylaimus* | Om4 | 1.2 | 1.8 | 1.0 | 0.9 | 0.8 | 0.4 | 0.0 |
| *Eudorylaimus* | Om4 | 0.2 | 0.2 | 0.4 | 0.0 | 0.2 | 0.0 | 0.0 |
| *Dorydorella* | Om4 | 5.5 | 6.7 | 4.5 | 4.1 | 3.8 | 0.4 | 0.5 |
| *Longidorella* | Om4 | 0.2 | 0.0 | 0.1 | 0.0 | 0.7 | 0.4 | 0.0 |
| *Aporcelaimium* | Om5 | 0.5 | 0.0 | 0.1 | 0.5 | 0.3 | 0.6 | 0.0 |
| *Campydora* | Om5 | 0.9 | 1.4 | 0.3 | 0.2 | 0.2 | 0.4 | 0.0 |
| *Axonchium* | Om5 | 0.6 | 0.5 | 0.4 | 0.6 | 0.8 | 1.2 | 0.2 |
| *Dorylaimellus* | Om5 | 4.5 | 5.6 | 3.6 | 5.8 | 6.0 | 2.1 | 2.0 |
| *Nygolaimus* | Ca5 | 0.0 | 0.8 | 0.7 | 0.0 | 0.2 | 0.0 | 0.0 |
| *Aetholaimus* | Ca5 | 0.2 | 2.7 | 0.1 | 0.0 | 0.0 | 0.0 | 0.0 |
