# Supplementary material for: Nitrogen Addition Regulates Soil Nematode Community Composition through Ammonium Suppression
Source: PLoS One. 2012 Aug 31;7(8):e43384. doi: 10.1371/journal.pone.0043384 (PMC3432042; doi:10.1371/journal.pone.0043384)
Supplement: Table S4 — Relative abundance of nematode genera (%) for Control and N addition treatments in September 2009. Data are mean values (N = 6). H = herbivores, Ba = bacterivores, Fu = fungivores, Om = omnivores, Ca = carnivores. (DOCX) [file pone.0043384.s008.docx]

Table S4. Relative abundance of nematode genera (%) for Control and N addition treatments in September 2009. Data are mean values (N = 6). H = herbivores, Ba = bacterivores, Fu = fungivores, Om = omnivores, Ca = carnivores.

| Genus | Guild |  | N addition level | | | | | | |
| --- | --- | --- | --- | --- | --- | --- | --- | --- | --- |
|  |  |  | Control | N_0_ | N_0.4_ | N_0.8_ | N_1.6_ | N_2.8_ | N_4.0_ |
| *Helicotylenchus* | H3 |  | 6.7 | 9.3 | 9.8 | 9.5 | 12.5 | 11.0 | 15.6 |
| *Pararotylenchus* | H3 |  | 2.9 | 3.0 | 3.4 | 4.0 | 5.2 | 4.4 | 4.3 |
| *Rotylenchus* | H3 |  | 0.3 | 0.3 | 0.6 | 0.2 | 0.8 | 0.9 | 1.0 |
| *Hemicriconemoides* | H3 |  | 0.0 | 0.0 | 0.3 | 0.0 | 0.3 | 0.0 | 0.2 |
| *Geocenamus* | H2 |  | 14.9 | 11.4 | 15.5 | 12.4 | 15.2 | 14.1 | 12.4 |
| *Amplimerlinius* | H2 |  | 0.2 | 0.2 | 0.0 | 0.0 | 0.0 | 0.0 | 0.6 |
| *Nagelus* | H2 |  | 3.7 | 2.2 | 3.7 | 3.7 | 2.3 | 2.4 | 4.8 |
| *Paralongidorus* | H5 |  | 0.2 | 0.0 | 0.3 | 0.3 | 0.3 | 0.3 | 0.3 |
| *Longidorus* | H5 |  | 0.3 | 0.8 | 0.0 | 0.2 | 0.0 | 0.8 | 0.2 |
| *Pratylenchus* | H3 |  | 0.0 | 0.0 | 0.0 | 0.0 | 0.3 | 0.9 | 0.3 |
| *Rhabditis* | Ba1 |  | 2.6 | 1.3 | 0.6 | 0.3 | 0.0 | 0.2 | 0.5 |
| *Cephalobus* | Ba2 |  | 1.1 | 1.1 | 1.0 | 1.9 | 2.5 | 6.9 | 5.1 |
| *Cervidellus* | Ba2 |  | 15.9 | 16.8 | 16.8 | 22.1 | 19.5 | 18.2 | 12.5 |
| *Acrobeles* | Ba2 |  | 7.1 | 11.1 | 8.3 | 6.5 | 7.4 | 5.2 | 4.0 |
| *Chiloplacus* | Ba2 |  | 0.2 | 0.5 | 0.0 | 0.8 | 1.2 | 1.5 | 2.5 |
| *Hetetorocephalobus* | Ba2 |  | 0.2 | 0.2 | 0.2 | 0.3 | 0.2 | 0.0 | 0.0 |
| *Acrobeloides* | Ba2 |  | 0.8 | 1.8 | 0.5 | 2.7 | 5.5 | 11.2 | 12.9 |
| *Eucephalobus* | Ba2 |  | 2.1 | 5.1 | 3.8 | 2.5 | 3.1 | 2.1 | 1.9 |
| *Acrobelophis* | Ba2 |  | 0.2 | 0.2 | 0.0 | 0.0 | 0.0 | 0.2 | 0.6 |
| *Wilsonema* | Ba2 |  | 0.0 | 0.0 | 0.0 | 0.2 | 0.5 | 0.0 | 0.0 |
| *Alaimus* | Ba4 |  | 2.2 | 1.6 | 1.0 | 1.0 | 0.8 | 0.6 | 0.0 |
| *Paramphidelus* | Ba4 |  | 0.0 | 0.0 | 0.3 | 0.2 | 0.0 | 0.2 | 0.0 |
| *Panagrolaimus* | Ba1 |  | 0.0 | 0.0 | 0.3 | 0.0 | 0.0 | 0.2 | 1.3 |
| *Boleodorus* | Fu2 |  | 0.2 | 0.3 | 0.2 | 0.0 | 0.0 | 0.6 | 0.2 |
| *Malenchus* | Fu2 |  | 1.6 | 1.1 | 1.3 | 1.6 | 1.1 | 0.3 | 0.5 |
| *Tylenchus* | Fu2 |  | 1.6 | 0.3 | 0.5 | 0.3 | 0.3 | 0.2 | 0.2 |
| *Filenchus* | Fu2 |  | 5.1 | 3.2 | 4.3 | 6.8 | 7.2 | 3.8 | 3.0 |
| *Paraphelenchus* | Fu2 |  | 1.3 | 1.6 | 1.9 | 1.9 | 2.0 | 2.0 | 2.1 |
| *Ditylenchus* | Fu2 |  | 0.0 | 0.2 | 1.6 | 0.3 | 0.3 | 0.5 | 1.1 |
| *Diphtherophora* | Fu3 |  | 0.5 | 0.2 | 0.5 | 1.4 | 1.1 | 0.9 | 0.5 |
| *Dorylaimoides* | Fu4 |  | 0.5 | 0.3 | 0.2 | 0.2 | 0.3 | 0.2 | 0.0 |
| *Tylencholaimus* | Fu4 |  | 0.6 | 1.4 | 1.0 | 1.3 | 0.3 | 0.9 | 0.3 |
| *Tylencholaimellus* | Fu4 |  | 8.5 | 7.4 | 5.8 | 5.6 | 2.3 | 1.4 | 2.4 |
| *Funaria* | Fu4 |  | 0.6 | 1.3 | 0.8 | 0.5 | 0.3 | 0.3 | 0.6 |
| *Thonus* | OM4 |  | 1.1 | 1.0 | 1.3 | 0.5 | 0.2 | 0.0 | 0.3 |
| *Microdorylaimus* | OM4 |  | 1.8 | 0.6 | 1.6 | 1.0 | 0.0 | 0.3 | 0.5 |
| *Eudorylaimus* | OM4 |  | 1.3 | 0.5 | 0.5 | 1.1 | 0.0 | 0.5 | 0.6 |
| *Dorydorella* | OM4 |  | 0.3 | 0.3 | 0.0 | 0.3 | 0.2 | 0.0 | 0.2 |
| *Aporcelaimellus* | OM5 |  | 0.8 | 1.0 | 0.8 | 0.5 | 0.0 | 0.0 | 0.0 |
| *Aporcelaimium* | OM5 |  | 0.6 | 0.2 | 0.2 | 0.2 | 0.0 | 0.5 | 0.3 |
| *Thornia* | OM4 |  | 0.6 | 0.6 | 0.3 | 0.3 | 0.2 | 0.0 | 0.2 |
| *Longidorella* | OM4 |  | 1.4 | 1.1 | 1.1 | 0.2 | 0.0 | 1.2 | 0.5 |
| *Campydora* | OM5 |  | 1.1 | 2.6 | 1.3 | 1.3 | 0.5 | 0.5 | 0.2 |
| *Discolaimus* | OM5 |  | 0.0 | 0.5 | 0.0 | 0.3 | 0.0 | 0.0 | 0.3 |
| *Carcharolaimus* | OM5 |  | 0.3 | 0.0 | 0.2 | 0.0 | 0.0 | 0.3 | 0.5 |
| *Axonchium* | OM5 |  | 0.5 | 1.0 | 0.6 | 0.5 | 0.5 | 0.0 | 0.5 |
| *Dorylaimellus* | OM5 |  | 6.7 | 5.4 | 6.7 | 3.7 | 5.1 | 4.3 | 4.3 |
| *Nygolaimus* | Ca5 |  | 1.1 | 1.0 | 1.0 | 1.7 | 0.8 | 0.3 | 0.2 |
| *Mylonchulus* | Ca3 |  | 0.3 | 0.2 | 0.0 | 0.0 | 0.0 | 0.0 | 0.0 |
